# Hepatopancreatoduodenectomy for local recurrence of cholangiocarcinoma after excision of a type IV-A congenital choledochal cyst: a case report

**DOI:** 10.1186/s40792-016-0146-5

**Published:** 2016-02-24

**Authors:** Mihoko Yamada, Tomoki Ebata, Gen Sugawara, Tsuyoshi Igami, Takashi Mizuno, Yuji Shingu, Masato Nagino

**Affiliations:** Division of Surgical Oncology, Department of Surgery, Nagoya University Graduate School of Medicine, 65 Tsurumai-cho, Showa-ku, Nagoya, 466-8550 Japan

**Keywords:** Hepatopancreatoduodenectomy, Biliary tract cancer, Surgery for recurrence

## Abstract

Surgical resection is the only curative treatment for biliary tract cancer (BTC); however, the recurrence rate remains high even after curative resection. There are limited data regarding the effectiveness of surgical resection for recurrent BTC. We report the favorable survival outcome of a patient who underwent a hepatopancreatoduodenectomy for local recurrence of cholangiocarcinoma after excision of a type IV-A congenital choledochal cyst. The patient, a 25-year-old woman, had undergone excision of a type IV-A congenital choledochal cyst with hepaticojejunostomy. The resected specimen revealed an early cholangiocarcinoma. The local recurrence at the site of anastomosis was detected 4 years and 4 months after surgery. We performed a left trisectionectomy with caudate lobectomy combined with hepatic artery and portal vein resections and a pancreaticoduodenectomy. Histological examination revealed a moderately differentiated adenocarcinoma, and the final diagnosis was recurrence of cholangiocarcinoma. There are a few reports of extensive resection for recurrence of BTC; however, aggressive surgery is possible and may offer favorable survival in selected patients.

## Background

Surgical resection is the only curative treatment for biliary tract cancer (BTC), including cholangiocarcinoma [1] and gallbladder carcinoma [2]. The 5-year survival rate is unsatisfactory even after curative resection: 33.1 % in cholangiocarcinoma and 41.6 % in gallbladder carcinoma [3]. The recurrence rate remains high and is reported to range from 46 to 71 % [4–6]. Most patients with recurrence undergo systemic chemotherapy [7]. However, the survival benefit is insufficient; Valle et al [7] reported in their trial that the median overall survival was only 11.7 months and no patients survived for more than 3 years. Recently, the effectiveness of surgical treatment for recurrence of BTC has been reported [4, 8, 9]. To our knowledge, few cases of recurrent BTC treated by hepatopancreatoduodenectomy (HPD) have been reported. Here, we report the favorable survival outcome of a patient who underwent an HPD combined with hepatic artery and portal vein (PV) resections for cholangiocarcinoma recurrence arising after a surgery for a type IV-A congenital choledochal cyst.

## Case presentation

A 25-year-old woman who was diagnosed with recurrence of cholangiocarcinoma after excision of a type IV-A congenital choledochal cyst was referred to our hospital for further evaluation and treatment. The patient had undergone excision of a congenital choledochal cyst with Roux-en-Y hepaticojejunostomy reconstruction 5 years prior. The pathological examination of the resected specimen revealed a well-differentiated tubular adenocarcinoma invading the fibromuscular layer in the cyst wall. Four years and 4 months after the excision, abdominal computed tomography (CT) revealed a mass at the hepatic hilum with narrowing of the hepaticojejunal anastomosis and duodenum. Percutaneous transhepatic biliary drainage was performed, but the cytology specimen was negative for malignant cells. Furthermore, although the patient underwent gastrojejunostomy because of duodenal stenosis, a specimen could not be taken from the mass; therefore, she was diagnosed with a recurrence of cholangiocarcinoma based on the laparotomy findings. The patient underwent a metallic stent placement at the narrow site of the anastomosis and systemic chemotherapy with gemcitabine. However, because of repeated bouts of cholangitis, the patient was transferred to our hospital for possible surgery.

When the patient was transferred, she presented with no symptoms. Liver function tests revealed the following slight abnormalities: total bilirubin, 0.7 mg/dl; aspartate aminotransferase, 23 IU/l; alanine aminotransferase, 16 IU/l; γ-glutamyl transpeptidase, 64 IU/l; and alkaline phosphatase, 225 IU/l. The serum carcinoembryonic antigen level was 1.4 IU/l, and the carbohydrate antigen 19-9 level was elevated to 255 IU/l. The plasma clearance rate of indocyanine green was 0.213.

**Fig. 1:**
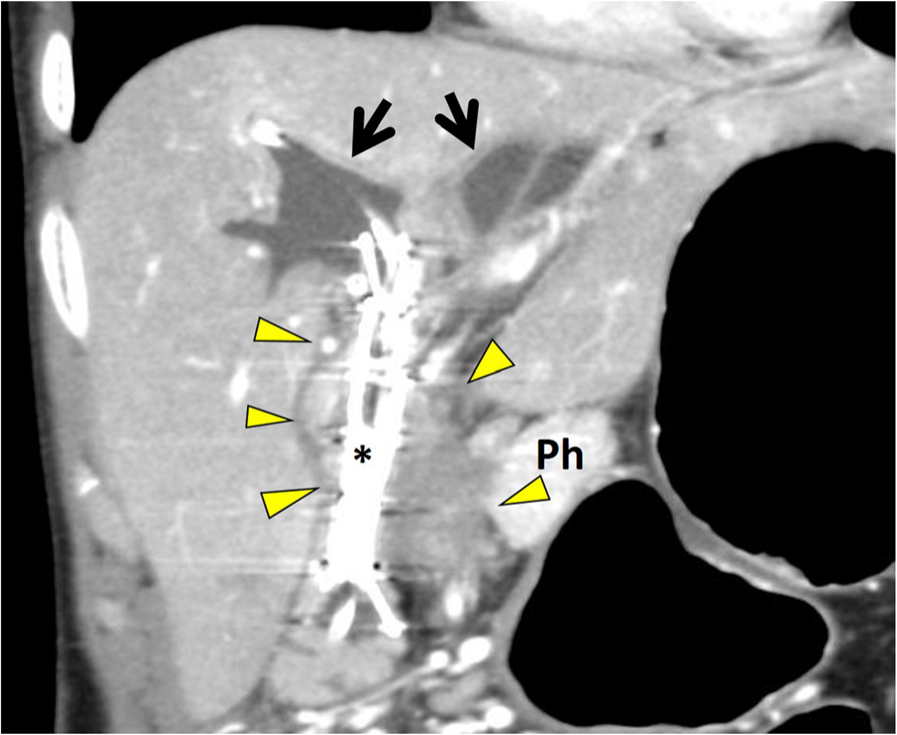
Computed tomography. A mass with an unclear and irregular border (*yellow arrow heads*) spreads from the hepatic hilum to the pancreatic head and invades the duodenum and the pancreatic head. The intrahepatic bile ducts were dilated (*black arrows*). *Ph* pancreatic head, *asterisk* metallic stent

**Fig. 2:**
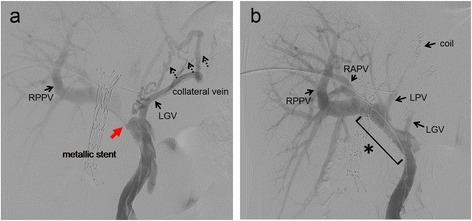
Portography. **a** Portal vein stenosis (*a red arrow*) with collateral veins from the left gastric vein are shown (*dotted black arrows*). **b** After performing a portal vein stenting and left portal vein embolization, the portal vein stenosis was resolved. *LGV* left gastric vein, *RAPV* right anterior portal vein, *RPPV* right posterior portal vein, *LPV* left portal vein, *asterisk* metallic stent placed in the portal vein

CT revealed a mass over the pancreatic head from the hepatic hilum with an unclear and irregular border invading the duodenum and pancreatic head (Fig. 1). The mass invaded from the main PV to the bifurcation of the right anterior and posterior PV. The main PV was severely strictured (Fig. 2a). The common hepatic artery, gastroduodenal artery, and right and left hepatic arteries were also involved by the mass.

**Fig. 3:**
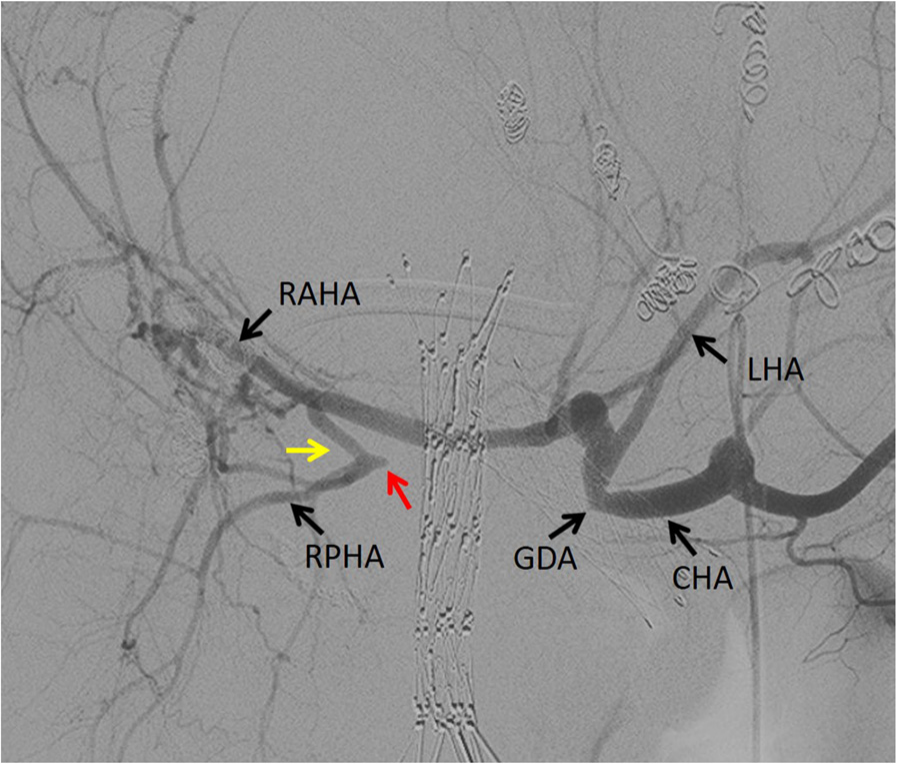
Angiography. The right posterior hepatic artery is disrupted at the hepatic hilum (*a red arrow*), and there is a collateral artery (*a yellow arrow*) from the right anterior hepatic artery to the right posterior hepatic artery. *CHA* common hepatic artery, *GDA* gastroduodenal artery, *LHA* left hepatic artery, *RAHA* right posterior hepatic artery, *RPHA* right posterior hepatic artery

Angiography revealed that the right posterior hepatic artery was invaded and disrupted at the hepatic hilum. Furthermore, a collateral artery from the right anterior hepatic artery to the right posterior hepatic artery was observed (Fig. 3).

**Fig. 4:**
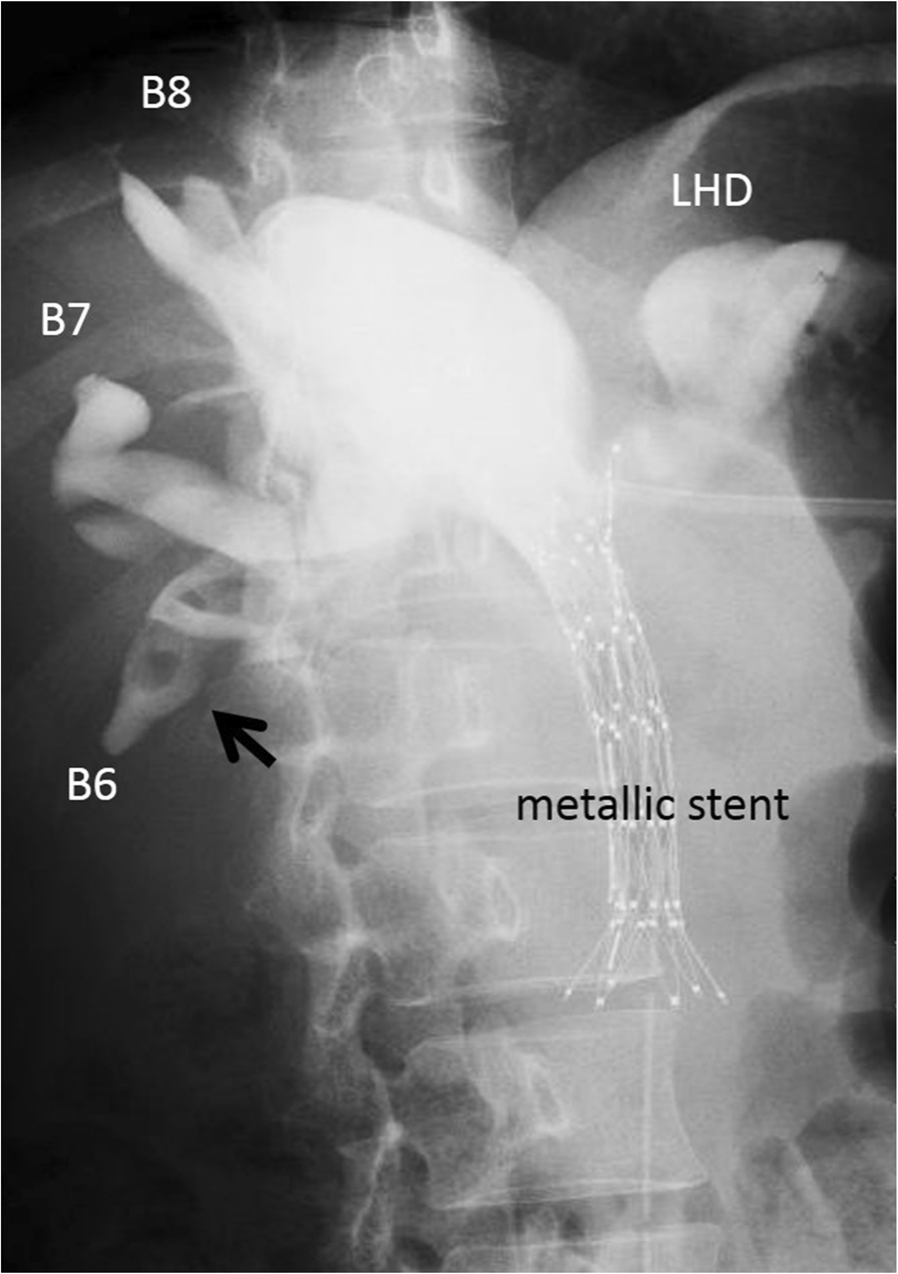
Cholangiography. The cystic dilation of the left hepatic duct (*LHD*), right anterior hepatic duct, and right hepatic duct are shown. There are intrahepatic calculi in the right posterior hepatic duct (*a black arrow*)

**Fig. 5:**
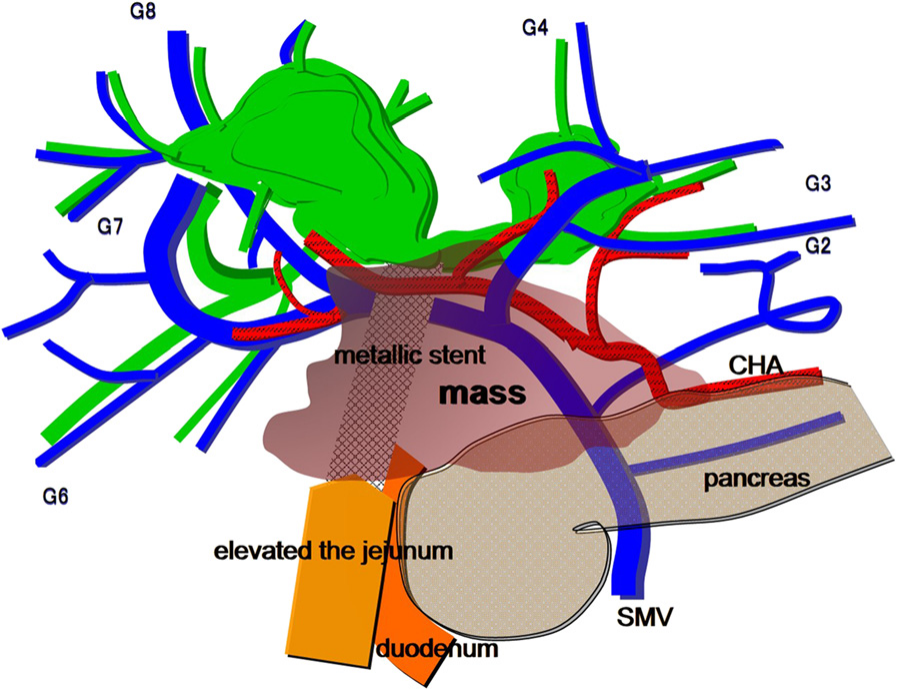
Preoperative schema. The mass involves the duodenum, pancreatic head, right hepatic artery, and main portal vein. *CHA* common hepatic artery, *SMV* superior mesenteric vein, *PV stent* portal vein stent

Cholangiography revealed cystic dilation of the left hepatic duct, right anterior hepatic duct, and right hepatic duct. There were intrahepatic calculi in the right posterior hepatic duct (Fig. 4). Bile cytology was negative. Although a histological diagnosis was unable to be made, according to these findings, we diagnosed this case as a recurrence of cholangiocarcinoma with invasion of the duodenum, pancreatic head, right hepatic artery, and main PV (Fig. 5). A left trisectionectomy with caudate lobectomy accompanied by right hepatic artery and PV resections and a pancreaticoduodenectomy (PD) were scheduled.

Although the future remnant liver function was sufficient, we preoperatively performed transhepatic PV stenting to reduce the flow within the collateral vein and left and right anterior PV embolizations (PVE) to increase liver function (Fig. 2b). Intrahepatic calculi were not removed before the surgery because of the high possibility of bleeding from the intrahepatic duct.

**Fig. 6:**
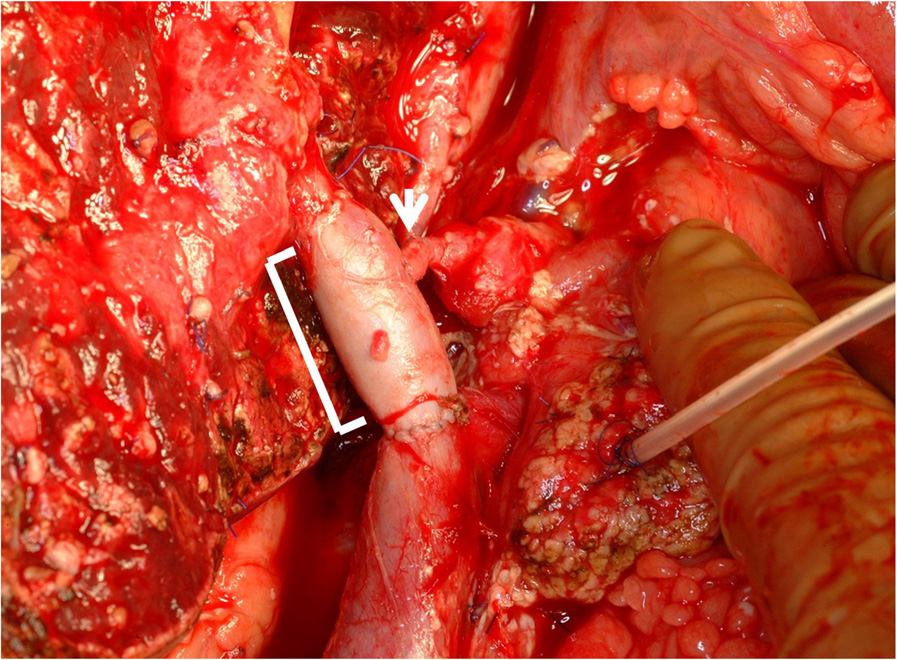
Completion photograph of hepatopancreatoduodenectomy. The *white bracket* indicates portal vein reconstruction using a right external iliac vein interposition grafting. The *white arrow* indicates arterio-portal anastomosis performed by anastomosing the common hepatic artery to the portal vein using a left radial artery interposition grafting

The patient then underwent an extended surgery as scheduled. Because of the infiltration of the transverse colon mesentery, the patient also underwent a partial colectomy. PV reconstruction was performed using a right external iliac vein interposition grafting. Because the patency of the right posterior hepatic artery failed to be confirmed, arterioportal shunting (APS) was performed by anastomosing the common hepatic artery to the PV using a left radial artery interposition graft (Fig. 6). The surgical time was 1167 min, and blood loss was 10,799 ml.

**Fig. 7:**
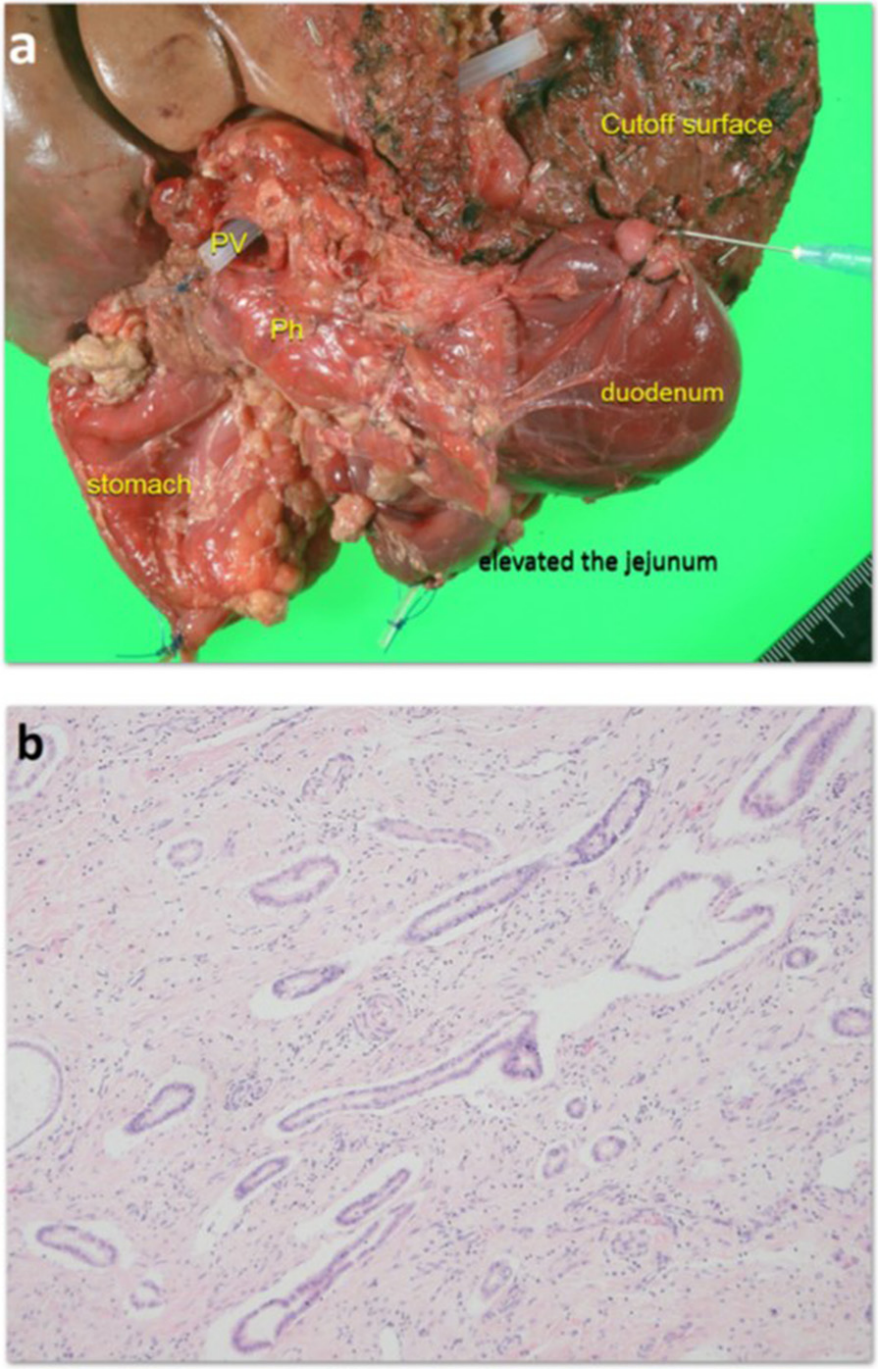
Macroscopic and histological findings. **a** Macroscopically, the mass invades the duodenum, jejunum, and pancreatic head. **b** Histological examination reveals a moderately differentiated adenocarcinoma. *PV* portal vein, *Ph* pancreatic head

The patient developed postoperative sepsis and a grade B (International Study Group of Pancreatic Surgery) pancreatic fistula. Both of these complications resolved through conservative therapy. The flow from the subphrenic artery to the remnant liver was confirmed on postoperative day 3, whereas the arterioportal anastomosis was spontaneously obstructed by postoperative day 24. Macroscopically, the mass at the hepatic hilum invaded the duodenum, jejunum, and pancreatic head (Fig. 7a). Histological examination revealed a moderately differentiated adenocarcinoma with infiltration of the pancreatic head, jejunum, and wall of the PV (Fig. 7b). The hepatic artery was surrounded by tumor cells. Thus, a diagnosis of cholangiocarcinoma recurrence was made. The patient was discharged on postoperative day 46 in good health.

The patient received postoperative systemic chemotherapy with gemcitabine. Two years and 6 months after surgery, a metastatic mass in the abdominal wall was revealed, and the patient underwent resection of the mass twice. However, a year after the last resection, the mass of the abdominal wall invaded the small intestine and was judged unresectable. The patient died from the primary disease 7 years and 2 months after HPD.

### Discussion

A few surgical studies for BTC recurrence have been reported. According to our search of the English literature, there are several case reports [5, 6, 10] and three retrospective cohort studies [4, 8, 9] pertaining to the surgical treatment of BTC. The three studies are as follows: Takahashi et al [4] performed surgery for recurrence in 74 (12.2 %) of the 606 patients with BTC. The surgeries for locoregional recurrence included nine hepatectomies with extrahepatic bile duct resections, vessel resections, and PDs, which were challenging and technically demanding surgeries. The survival after recurrence in the resection group was significantly better than that in the non-resection group (survival rate, 37 % vs. 3 % at 3 years and 14 % vs. 0.3 % at 5 years; *P* < 0.001). Song et al. [8] performed surgery for recurrence in 27 (8.5 %) of the 316 patients with BTC, including 7 hepatectomies, 8 PDs, and 12 other surgeries. The authors also reported significantly better survival after recurrence in cases of surgical treatment (median survival time after recurrence, 18.9 vs. 7.7 months; *P* < 0.001). Noji et al. [9] performed surgery for recurrence in 27 (18 %) of the 150 patients with gallbladder carcinoma and extrahepatic cholangiocarcinoma, including 18 hepatectomies (7 major and 11 non-anatomical hepatectomies), 1 non-anatomical hepatectomy and inferior venous cava/jejunum/colon/diaphragm resection, 2 PDs, and 9 other surgeries. The authors reported that survival in patients with resection was significantly better than in those without resection (overall cumulative 5-year survival rate, 23.6 % vs. 0 %; median survival time, 21.6 vs. 9.5 months; *P* < 0.01).

Thus, three studies concluded that resection for recurrence of BTC in selected patients (8.5–18 % of their recurrent patients) was feasible and offered survival benefits. Additionally, a few patients, as in our case, exhibited a greater than 5-year survival after surgery. With respect to the types of surgery, most of the surgeries were simple resections, such as partial hepatectomy and mastectomy, and challenging surgeries, like that in our case, were extremely rare. Surgery for locoregional recurrence requires extended resection, including adjacent significant organs and vessels to achieve R0 resection, and demands a high degree of skill. Therefore, surgical indication should be carefully determined.

HPD for BTC is one of the most challenging surgeries, and even today, the surgery is associated with high morbidity; however, the low mortality and survival benefits of the surgery combined with PV and/or artery resection has been described [11]. HPD for recurrence of BTC is rare, and only two cases have been reported previously [12, 13]. In both of the two reported cases, HPD was successfully performed without vessel resection in patients with locoregional recurrence of cholangiocarcinoma. In our case, intending to a curative resection, it was assumed that HPD combined with vessel resection was required because the mass had infiltrated into the adjacent organs, right hepatic artery, and PV.

Before extended hepatectomy, PVE has been reported to enlarge the future liver remnant and improve the liver function [14]. However, PV stenosis, as occurred in our case, prevents this process. Therefore, PVE with PV stenting was performed. Preoperative PVE with PV stenting was reported to be effective in a case of severe PV tumor invasion and stenosis, thus enabling extended hepatectomy to be performed [15].

The patient was scheduled for arterial reconstruction combining the common hepatic artery and the right posterior hepatic artery. However, because the patency of the right posterior hepatic artery was not able to be confirmed, APS was used. Some reports indicate that APS is a feasible and safe alternative to the reconstruction of hepatic arteries [16, 17]. A few complications after APS, such as bile leakage and liver abscess, have also been reported. Fortunately, in our case, because blood flow came from the subphrenic artery to the remnant liver, there were no serious complications.

## Conclusions

By undertaking careful perioperative management, we successfully performed a curative resection in a patient with cholangiocarcinoma recurrence, even though there was vascular infiltration.

## Consent

Written informed consent was obtained from the parents of the patient for publication of this case report and any accompanying images. A copy of the written consent is available for review by the Editor-in-Chief of this journal.

## Abbreviations

APS: arterioportal shunting
BTC: biliary tract cancer
CT: computed tomography
HPD: hepatopancreatoduodenectomy
PD: pancreaticoduodenectomy
PV: portal vein
PVE: portal vein embolization
